# Effects of Structure and Composition of Adsorbents on Competitive Adsorption of Gaseous Emissions: Experiment and Modeling

**DOI:** 10.3390/nano13040724

**Published:** 2023-02-14

**Authors:** Adam Verner, Jonáš Tokarský, Tomáš Najser, Lenka Matějová, Kateřina Mamulová Kutláková, Jan Kielar, Václav Peer

**Affiliations:** 1 Nanotechnology Centre, CEET, VSB—Technical University of Ostrava, 17. listopadu 2172/15, 708 00 Ostrava-Poruba, Czech Republic; 2 ENET Centre, CEET, VSB—Technical University of Ostrava, 17. listopadu 2172/15, 708 00 Ostrava-Poruba, Czech Republic; 3 Faculty of Materials Science and Technology, VSB—Technical University of Ostrava, 17. listopadu 2172/15, 708 00 Ostrava-Poruba, Czech Republic; 4 Institute of Environmental Technology, CEET, VSB—Technical University of Ostrava, 17. listopadu 2172/15, 708 00 Ostrava-Poruba, Czech Republic

**Keywords:** gaseous emission, competitive adsorption, molecular modeling, morphology, adsorbent structure

## Abstract

Dangerous gases arising from combustion processes must be removed from the air simply and cheaply, e.g., by adsorption. This work is focused on competitive adsorption experiments and force field-based molecular modeling of the interactions at the molecular level. Emission gas, containing CO, NO, SO_2_, and CO_2_, was adsorbed on activated carbon, clay mineral, silicon dioxide, cellulose, or polypropylene at two different temperatures. At 20 °C, activated carbon had the highest NO and SO_2_ adsorption capacity (120.83 and 3549.61 μg/g, respectively). At 110 °C, the highest NO and SO_2_ adsorption capacity (6.20 and 1182.46 μg/g, respectively) was observed for clay. CO was adsorbed very weakly, CO_2_ not at all. SO_2_ was adsorbed better than NO, which correlated with modeling results showing positive influence of carboxyl and hydroxyl functional groups on the adsorption. In addition to the wide range of adsorbents, the main novelty of this study is the modeling strategy enabling the simulation of surfaces with pores of controllable sizes and shapes, and the agreement of the results achieved by this strategy with the results obtained by more computationally demanding methods. Moreover, the agreement with experimental data shows the modeling strategy to be a valuable tool for further adsorption studies.

## 1. Introduction

In recent years, more than ever before, the importance of monitoring the evolution of the amount of pollutants in the environment has increased [1]. People and nature in general need optimal conditions for their efficient functioning, which is related to the reduction of destructive factors and the increase of constructive factors in the environment. Manufacturing industry is accompanied by the disruption of natural resources during the acquisition of input materials, a number of chemical reactions or energy processes during production, and last but not least, the disposal of waste, i.e., unused or unusable products. The main sources of pollution include the manufacturing and processing industry [2] as well as the energy industry [3] and transportation [4], with which the increasing production of specifically air pollutants is connected.

Despite the fact that thermochemical conversion is beginning to be replaced by the use of renewable resources worldwide, the combustion process of various materials (coal, gas, heating oils, wood, biomass, etc.) still contributes significantly to air pollution [5,6]. Even with the effective use of plant waste [7,8], it is still challenging to monitor and reduce the risks related to the production of arising pollutants. While solid pollutants consist primarily of particulate matter of various grain sizes and NH_3_, gaseous pollutants mainly include CO_2_, CO, SO_2_, nitrogen oxides (NO_x_) [9,10], volatile organic compounds and polycyclic aromatic hydrocarbons [6,10]. Solid particles released during the combustion process can be easily captured by various types of solid or liquid filter systems. However, the removal of emission gases from the air is generally more complex, due to the gaseous state of the pollutants, the required chemical selectivity, etc. The most studied is CO_2_ removal from the air [11]. Absorption of CO_2_ in gas-liquid systems with methanol, poly-(ethylene glycol) dimethyl ether [12] or amine solutions [13] as absorbing phases was investigated. However, adsorption methods in gas-solid systems, i.e., weaker physisorption or stronger chemisorption [11,14,15], are used more often. The most effective adsorption materials for CO_2_ include activated carbons [16,17], zeolites [18], porous oxides (e.g., calcium oxides [19] or silica gel [20]), hydrotalcites [21], amines [22] or metal-organic frameworks [11,23,24].

On the other hand, the adsorption mechanisms and suitable adsorbents of NO_x_, CO, and SO_2_ have been considerably less investigated. Research on CO and NO adsorption using Pd catalysts [25], possibly Au alloys [26] or various modifications of activated carbon [27] should be mentioned. In the case of SO_2_ and NO, adsorption mechanisms on modified activated carbon [27,28] and bimetallic surfaces containing noble and transition metals [29] were investigated. Zeolites are also efficient adsorbents of CO_2_, SO_2_, and NO [30].

Research works mostly deal with specific adsorption materials for a specific gas, but materials in various forms sorbing several types of emission gases at the same time by the mechanism of competitive adsorption have not yet been sufficiently investigated. There is also a lack of research on interactions at the molecular level during the competitive adsorption of emission gases. Therefore, in this work, the process of competitive adsorption of emission gases on different materials with different physical and chemical properties was studied both experimentally and via force field-based molecular modeling. Activated carbon is extensively researched and modeled in connection with adsorption [31,32,33,34]. Therefore, it was used for comparing the obtained data and verifying the modeling strategy. Adsorbent materials whose potential in the field of competitive adsorption of emission gases has not yet been established were used, and adsorption capacities were determined. Unlike DTF modeling methods that are usually used, the modeling strategy introduced here enabled the creation of larger models with pores of precisely controllable sizes and shapes. The influence of chemical and structural factors on competitive adsorption was clarified, which can be used further in research on other materials or as a prediction tool of adsorption.

## 2. Materials and Methods

### 2.1. Materials

Adsorption experiments were carried out using a special emission gas (EG) having concentrations of emission components similar to the real concentrations produced during the combustion of various alternative fuels, such as biomass, sewage sludge, etc. [35]. The EG consisted of 197.3 vol. ppm NO (199.9 *w*/*w* ppm), 197.3 vol. ppm SO_2_ (426.8 *w*/*w* ppm), 395 vol. ppm CO (373.6 *w*/*w* ppm), and 9.96 vol. % CO_2_ (14.80 *w*/*w* %) in nitrogen (SIAD Czech s.r.o., Ostrava, Czech Republic). In addition, the nitrogen was also used to purge the adsorption path.

The following materials commonly used in hazardous chemical spill incidents were used as adsorbents in this study: activated carbon (AC; MA C6 D40 CZ, Resorbent, s.r.o., Ostrava, Czech Republic), clay (CL; Bentonite Puranit, LIPERA s.r.o., Velke Bilovice, Czech Republic), and (from REO AMOS s.r.o., Ostrava, Czech Republic) silicon dioxide (SiO_2_), cellulose (CEL) and polypropylene (PP).

### 2.2. Characterization of Samples

A photoelectron spectrometer with a hemispherical VG SCIENTA R3000 analyzer (Prevac, Rogów, Poland) was used for XPS analysis of AC. The photoelectron spectra were recorded using a monochromatized aluminum Al_Kα_ source (*E* = 1486.6 eV) and a low-energy electron flood gun (FS40A-PS). The base pressure in the analysis chamber was 5·10^−9^ mbar. Spectra were recorded with a constant pass energy of 100 eV for the survey and high-resolution spectra. The binding energy scale was calibrated using the Au 4f7/2 line of a cleaned gold sample at 84.0 eV. The composition and chemical surrounding of the sample surface were investigated on the basis of the areas and binding energies of O 1s and C 1s photoelectron peaks. The fitting of high-resolution spectra was performed using CasaXPS software.

The moisture content of the samples was determined by Kern ABT 320-4NM (Kern & Sohn GmbH, Balingen, Germany). Particle density was determined by measuring the volume of the sample in water and the weight with MA 110.R laboratory scale (RADWAG Váhy s.r.o., Šumperk, Czech Republic). Atmospheric pressure and pressure in the adsorption system were measured with GPB 3300 (GHM-Greisinger s.r.o., Regenstauf, Germany) and Kimo MP 210 (Kimo Instruments, Mumbai, India), respectively. The concentration of NO, SO_2_ and CO was measured by the infrared spectrometer PG-350 (Horiba, Palaiseau, France). Nitrogen physisorption at 77 K was measured using 3Flex apparatus (Micromeritics, Norcross, CA, USA). Prior to physisorption analysis, each material was degassed under pressure of 0.6 bar and temperature of 350 °C. This step was done for all materials to release physisorbed moisture. After such pre-treatment, the nitrogen adsorption–desorption isotherms at 77 K of all materials were measured for the relative pressure range *p*/*p_0_*~1 × 10^−7^–0.99. Nitrogen adsorption–desorption data were processed using BET theory, the t-plot method and the BJH method applied on the adsorption branch of the nitrogen adsorption–desorption isotherm, by using either the Carbon Black STSA standard isotherm (for carbonaceous materials) or Broekhoff-De Boer standard isotherm (for inorganic materials). The slit or cylindrical-pore geometry of mesopores and macropores characterized by pore width or diameter, respectively, were assumed. From measured data, it was possible to reliably obtain the specific surface area, *S_BET_* (m^2^/g), the mesopore–macropore surface area, *S_meso_* (m^2^/g), the micropore volume, *V_micro_* (mmliq^3^/g) and mesopore–macropore size distribution characterized by the pore width or diameter. The net pore volume, *V_net_* (mm^3^liq/g), was evaluated from the adsorbed amount of nitrogen at *p*/*p_0_*~0.99. The micropore size distribution was evaluated from the adsorbed amount of nitrogen at *p*/*p_0_*~1 × 10^−7^–0.05 using the Horwath–Kawazoe solution and assuming the slit-pore geometry for both carbonaceous and inorganic materials.

X-ray powder diffraction (XRPD) patterns were recorded under Co_Kα_ irradiation (*λ* = 0.1789 nm, *U* = 35 kV, *I* = 25 mA) using the Bruker D8 Advance diffractometer (Bruker AXS, Karlsruhe, Germany) equipped with a fast position sensitive detector VÅNTEC 1. Measurements were carried out in the reflection mode, powder samples were pressed in a rotational holder, and a goniometer was used with the Bragg–Brentano geometry in 2θ range from 3 to 80°, step size 0.03°. The phase composition was evaluated using database PDF 2 Release 2020 (International Centre for Diffraction Data).

### 2.3. Sample Preparation

The powder samples (AC, CL, SiO_2_) were crushed and sieved into three granulometric fractions—0.16–0.315 mm, 0.315–0.63 mm, 0.63–1.00 mm (Table 1)—to ensure laminar flow in the adsorption chamber (Ad) whose inner tube diameter was 10 mm. CEL and PP were used in their original form, i.e., pellets and nonwoven, respectively. Each type of sample was used in its wet and dried state. The samples were dried in a drying oven Binder FP 400.

Particle density and humidity of the adsorbents were determined, and the samples were labeled according to the granulometric fraction (Table 1).

### 2.4. Adsorption System

**Figure 1:**
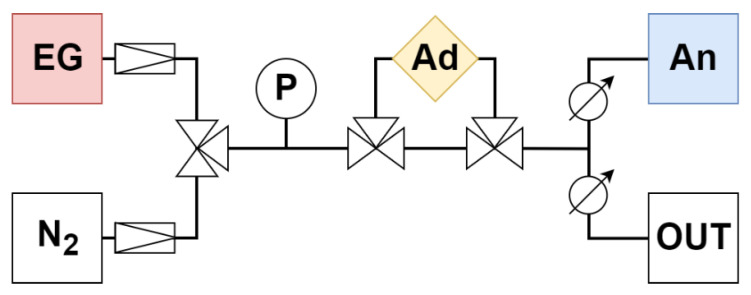
Diagram of adsorption apparatus with fittings. EG—emission gas, N_2_—nitrogen, P—pressure gauge, Ad—adsorption chamber, An—analyzer, OUT—overflow branch.

The adsorption experiments took place in a closed system consisting of PTFE hoses, stainless steel fittings, the stainless steel Ad and analytical instruments (Figure 1). The Ad was a stainless steel tube with an inner diameter of 10 mm. A volume of 3 cm^3^ was calculated from the volume of the catalyst *V_cat_* according to the formula:

V_cat_ = Q_V_/GHSV (1)

where *Q_V_* is volumetric gas flow rate per hour (0.5 L/min = 30,000 cm^3^/h) and *GHSV* is gas hourly space velocity (10,000 h^−1^) [36].

The gas traveled from the gas cylinders (EG—emission gas, N_2_—nitrogen) through the pressure gauge (P) and the adsorbent (Ad) to the analyzer (An) at a speed of 0.5 L/min (analyzer parameter). The rest was discharged through the overflow branch (OUT) (Figure 1).

First, the entire adsorption apparatus was filled with nitrogen (including the Ad branch). Subsequently, the source gas was switched from N_2_ to EG through the branch without Ad. The measurement of the concentration of emission components with the analyzer was switched on. As soon as the concentration values stabilized, thereby determining the actual concentrations of gaseous components in EG, the gas path was switched to the Ad branch, where adsorption took place. The measurement was terminated after the initial concentration was again reached (the actual concentration in the EG bottle). The measurement took place at 20 °C (with wet adsorbents) and 110 °C (with dried adsorbents and Ad in the drying oven Binder FP 400 (Binder GMBh, Tuttlingen, Germany). Each sample was measured three times and the results were averaged.

The total amount of each adsorbed gaseous component was obtained by integrating the area between the adsorption curve and the original concentration value of the given emission component, and using the ideal gas equation (where the amount of substance *n* is expressed as *m*/*M*) for expressed mass *m* of the adsorbed gas component:

*m* = *p*·*V*·*M*/(*R*·*T*) (2)

where *p* (Pa) is pressure in the adsorption system, *V* (dm^3^) is volume of the adsorbent, *M* (g·mol^−1^) is molar mass of the gaseous component, *R* (J·K^−1^·mol^−1^) is the gas constant, and *T* (K) is temperature in the adsorption system [37].

Adsorbed mass *m* was related to one gram of wet or dried adsorbent expressed in μg/g.

### 2.5. Atomistic Models and Modeling Strategy

**Figure 2:**
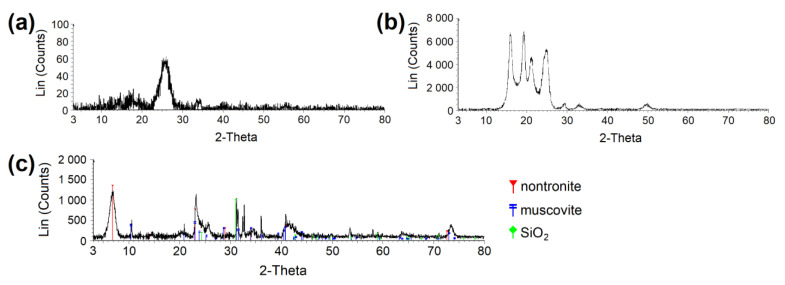
XRPD diffractogram proving the crystalline phase in (**a**) CEL and (**b**) PP. (**c**) Determination of clay phases in clay material. CEL—cellulose, PP—polypropylene.

Building of the initial models of EG molecule and adsorbent surface, geometry optimizations, molecular dynamics, and energy calculations were carried out in Biovia Materials Studio 7.0 (MS) modeling environment [38]. Triclinic unit cells of CEL (a = 8.549 Å, b = 9.568 Å, c = 10.000 Å, α = 102.10°, β = 106.54°, γ = 55.46°), and PP (a = 8.348 Å, b = 13.505 Å, c = 10.000 Å, α = 75.68°, β = 39.54°, γ = 61.01°), both containing two polymer chains, were created according to Chen et al. [39] and Verenich et al. [40], respectively (Figure 2).

**Figure 3:**
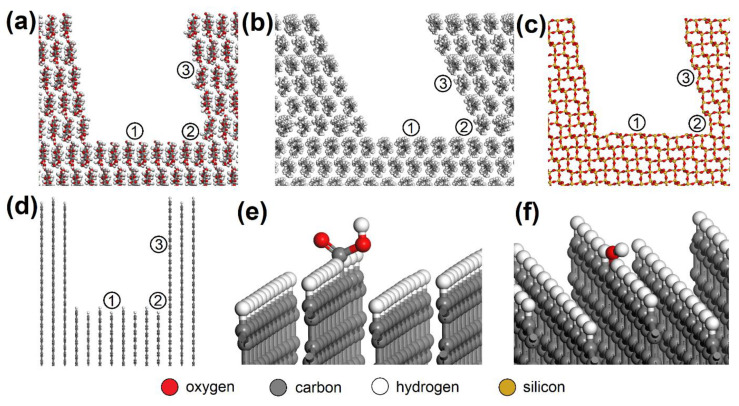
Nanopores in the (**a**) CEL, (**b**) PP, (**c**) SiO_2_, and (**d**) AC surfaces with spots where the NO or SO_2_ molecule was placed: (1) bottom, (2) corner, (3) side. NO or SO_2_ molecules were also placed on the AC surfaces with -COOH or -OH groups added, e.g. (**e**) bottom_COOH and (**f**) bottom_OH. CEL—cellulose, PP—polypropylene, SiO_2_—silicon dioxide, AC—activated carbon.

**Figure 4:**
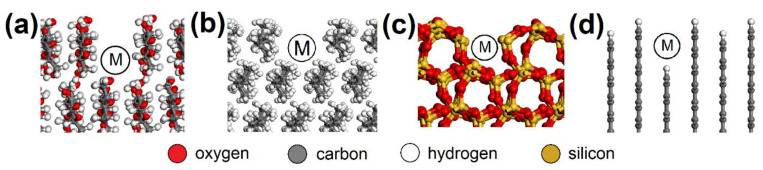
Molecular pores in the (**a**) CEL, (**b**) PP, (**c**) SiO_2_, and (**d**) AC surfaces with spots (M) where the NO or SO_2_ molecule was placed. CEL—cellulose, PP—polypropylene, SiO_2_—silicon dioxide, AC—activated carbon.

AC orthorhombic unit cell (a = b = 2.460 Å, c = 6.670 Å, α = β = 90°, γ = 120°) was prepared according to Wyckoff [41]. SiO_2_ trigonal (a = b = 4.913 Å, α = β = 90°, γ = 95.18°) and MUS monoclinic unit cells (a = 5.194 Å, b = 8.996 Å, c = 20.096 Å, α = β = 90°, γ = 120°, layer thickness ~6.7 Å, interlayer distance ~3.3 Å) were imported from the MS database. Substitutions and interlayer cations were added to MUS so that MUS and NON unit cells met the chemical formulas $\left(K_{0.86}Na_{0.10}\right)\left(Al_{1.90}Fe_{0.05}^{2+}Mg_{0.06}Fe_{0.02}^{3+}Ti_{0.01}\right)\left(Si_{3.02}Al_{0.98}\right)O_{10}{\left(OH\right)}_{1.99}F_{0.01}$ and $\left(Ca_{1.50}Na_{0.5}\right)\left(Fe_{1.84}^{3+}Al_{0.15}Mg_{0.02}{□}_{0.99}\right)\left(Si_{3.46}Al_{0.38}Fe_{0.16}^{3+}\right)O_{10}{\left(OH\right)}_{2}$, respectively, according to Weiss et al. [42]. Chemical formulas of prepared MUS and NON models were $\left(K_{180}Na_{18}\right)\left(Al_{334}Fe_{9}^{2+}Mg_{11}Fe_{4}^{3+}Ti_{2}\right)\left(Si_{540}Al_{180}\right)O_{1800}{\left(OH\right)}_{358}F_{2}$ and $\left(Ca_{270}Na_{90}\right)\left(Fe_{330}^{3+}Al_{25}Mg_{5}{□}_{180}\right)\left(Si_{620}Al_{70}Fe_{30}^{3+}\right)O_{1800}{\left(OH\right)}_{360}$, respectively. CEL, PP, SiO_2_, and AC periodic unit cells were enlarged to ~50 × 50 Å with a surface height and additional vacuum slab height of 50 Å and 350 Å, respectively. The CEL, PP, SiO_2_, and AC surfaces were further modified to simulate nanopores with dimensions of (width × height) ~30 × 30 Å (Figure 3) and molecular pores with dimensions of 5.1 × 7.1 Å (CEL), 6.5 × 5.9 Å (PP), 5.6 × 5.7 Å (SiO_2_), and 5.9 × 6.3 Å (AC) (Figure 4).

Due to the XPS analysis of AC adsorbents establishing 3.67 % carboxyl and 4.61 % hydroxyl groups on the surface, models with -COOH (bottom_COOH, corner_COOH, cavity_COOH) and -OH (bottom_OH, corner_OH, cavity_OH) added to the bare AC surfaces (bottom, corner, cavity) were also prepared (Figure 3e,f). The main advantage of using this approach to building AC models based on a graphitic structure is the possibility it offers of creating pores and cavities of controllable sizes [43].

**Figure 5:**
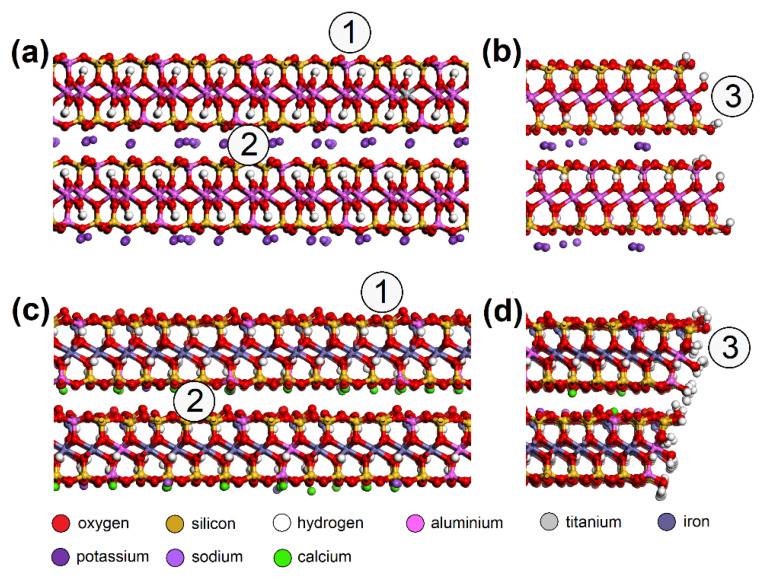
Molecular models of MUS (**a**) surface, (**b**) edge; and NON (**c**) surface and (**d**) edge—with spots where the NO or SO_2_ molecule was placed: (1) surface, (2) interlayer, (3) edge. MUS—muscovite, NON—nontronite.

MUS and NON periodic unit cells were enlarged to ~50 × 50 Å with a surface containing two clay layers with two interlayers and a vacuum slab added with a height of 350 Å. NO and SO_2_ molecules were placed on various spots of the surfaces (Figure 5). Three models were created for each “EG molecule/adsorbent surface spot” combination.

An MS/Forcite module was used for geometry optimization of each model. The COMPASS force field parameterized atoms and assigned their charges [44], which was verified for CEL [45], PP [46], SiO_2_ [47], and AC [43]. A universal force field, with the QEq module setting charges, was used for models containing MUS and NON [48].

The Smart algorithm (implemented in MS) with 5·10^5^ steps was used. Convergence thresholds for displacement, force, and energy were 5·10^−5^ Å, 5·10^−3^ kcal·mol^−1^·Å^−1^, and 1·10^−4^ kcal·mol^−1^, respectively. Cell parameters were not optimized. Interaction energy (*E_int_*; kcal/mol) was calculated for each optimized model from potential energies (*E_p_*) using the following equation:

*E_int_* = *E*_*p*1_ − *E*_*p*2_ − *E*_*p*3_ (3)

where *E_p_*_1_ is *E_p_* of a whole model, *E_p_*_2_ is *E_p_* of a surface, and *E_p_*_3_ is *E_p_* of a NO/SO_2_ molecule. The lower the *E_int_* value was, the stronger the interaction between NO/SO_2_ and the surfaces.

For molecular dynamics, periodic models of AC, MUS, and NON with dimensions of ~17 × 17 Å (α = β = 90°) were prepared. The AC model contained three graphene layers, while the MUS and NON models contained two clay layers with one interlayer. All models had a vacuum slab, 1000 Å high, perpendicular to the surfaces. A total of 28 NO and 28 SO_2_ molecules were evenly placed on one side of the surfaces. The MD/Forcite module and COMPASS or Universal force field for AC or MUS/NON models, respectively, were used for molecular dynamics with NVT ensemble, Nosé thermostat, random initial velocities, 293 K temperature, and 5 ns total simulation time.

## 3. Results and Discussion

### 3.1. Physisorption

**Figure 6:**
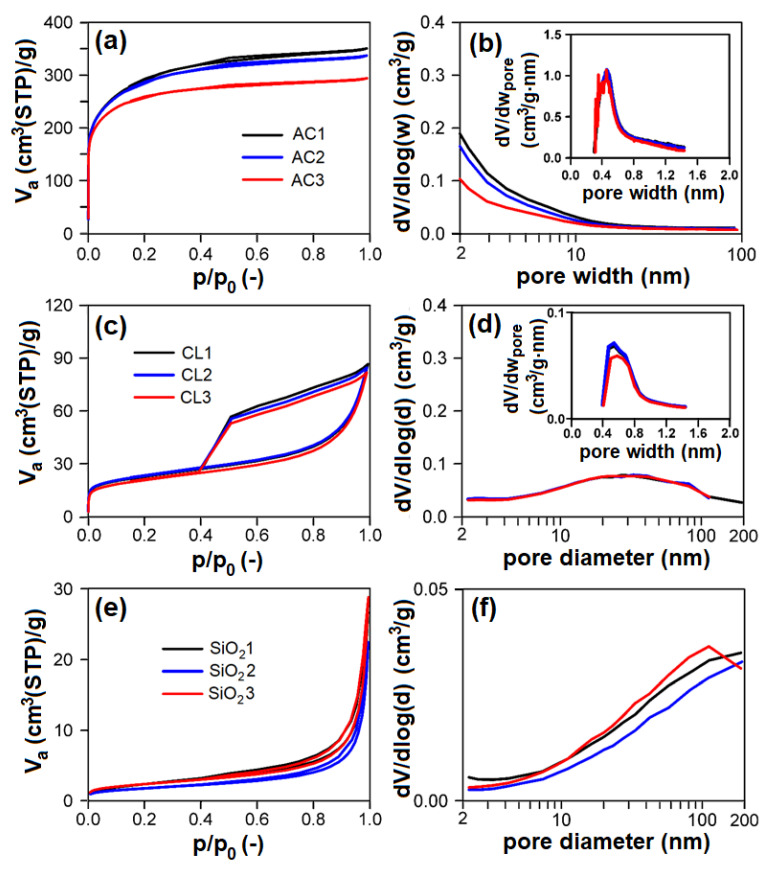
(left: (**a**,**c**,**e**)) Measured nitrogen adsorption–desorption isotherms at 77 K, (right: (**b**,**d**,**f**)) evaluated mesopore–macropore-size distributions of adsorbents and (inset) evaluated micropore size distributions of adsorbents: (**a**,**b**) AC; (**c**,**d**) CL; (**e**,**f**) SiO_2_. AC1-3, CL1-3, SiO_2_1-3—activated carbon, clay, and silicon dioxide respectively (1-3—three granulometric fractions; see Table 1), *V_a_*—adsorbed volume of nitrogen, STP—standard temperature and pressure, *p/p*_0_—relative pressure, *dV/dlog(w)*—derivation of adsorbed volume of nitrogen divided by derivation of logarithm of pore width, *dV/dw_pore_*—derivation of adsorbed volume of nitrogen divided by derivation of pore width, *dV/dlog(d)*—derivation of adsorbed volume of nitrogen divided by derivation of logarithm of pore diameter.

Nitrogen physisorption measurements (Figure 6a,c,e; Table 2) proved that individual groups of adsorbents differ very much concerning their textural properties. According to the surface area and porosity, the adsorbents used can be ordered as follows (from largest to smallest): AC > CL > SiO_2_ > CEL > PP.

AC showed the type I adsorption isotherm according to IUPAC classification [49], typical for microporous materials (Figure 6a). Evaluated textural parameters (Table 2) and pore-size distributions (Figure 6b and insets) definitively correspond to this feature. AC showed micropores of 0.48 nm width with a volume of 359–376 mm^3^liq/g, and mesopore surface area in the range of 114–209 m^2^/g. With increasing particle-size fraction in the range of 0.16–1 mm, the mesopore surface area and net pore volume of AC logically decreases with no effect on micropore volume.

CL showed the mixed I + IV type of adsorption isotherm (according to IUPAC classification) typical for mesoporous materials with broad mesopores/macropores and some micropores (Figure 6c,d). CL possessed ~14 mm^3^liq/g of micropores of ~0.6 nm width (Figure 6d inset) and broad mesopores/macropores of 47–50 m^2^/g surface area (Figure 6d). There are no differences between individual particle-size fractions of CL, the properties of individual particle-size fractions being identical.

Concerning SiO_2_, these samples show the type III adsorption isotherm (according to IUPAC classification) with narrow steep hysteresis loop (Figure 6e), typical for macroporous/nonporous materials with large macropores, which corresponds to the pore-size distributions shown in Figure 6f, proving the presence of macropores having diameter >100 nm. Textural properties (i.e., pore surface area, net pore volume) of SiO_2_ are comparable; there are no differences among particle-size fractions. PP and CEL belong among nonporous materials with very low surface area.

### 3.2. Emission Gas Adsorption

With the exception of PP, all adsorbent materials were able to adsorb at least one of the EG components (Table 3). CO_2_ was adsorbed on none of the adsorbents. Adsorption of CO on SiO_2_ was not observed at any temperature. Granulometry had a significant effect on the adsorbed amount of gases—the smaller the particles, the more adsorbed EG. At the adsorption temperature of 20 °C, AC on average adsorbed the most amount of both NO (9× higher than CL, 20× higher than CEL; Table 3) and SO_2_ (1.5× higher than CL, 3× higher than CEL, and 54× higher than SiO_2_; Table 3). At the adsorption temperature of 110 °C, all of the adsorbents adsorbed NO in very small amounts in contrast to SO_2_, which was adsorbed significantly more on average, and most on CL (6× higher than on AC, 7× higher than on CEL and 82× higher than on SiO_2_; Table 3). CEL was the only one to adsorb CO at 20 °C (33.55 μg/g). on average, CL adsorbed the most amount of CO at 110 °C.

**Figure 7:**
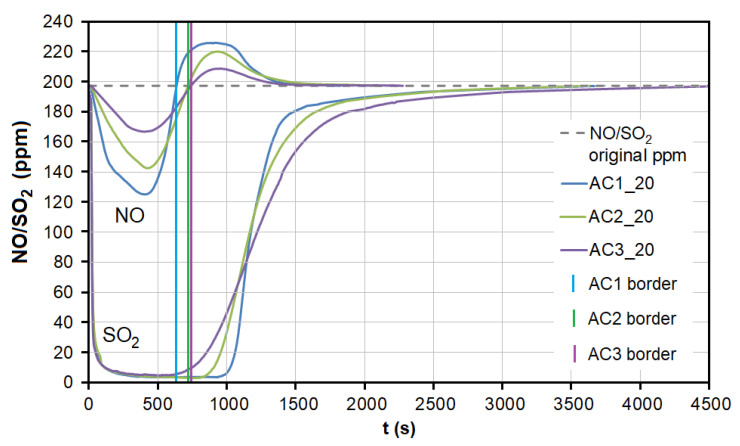
The dependence between SO_2_ adsorption and NO desorption at the temperature of 20 °C. AC1, AC2, and AC3 border lines represent the beginning of the NO desorption effect for each adsorbent. AC1-3—activated carbon at different granulometric fractions (see Table 1).

**Figure 8:**
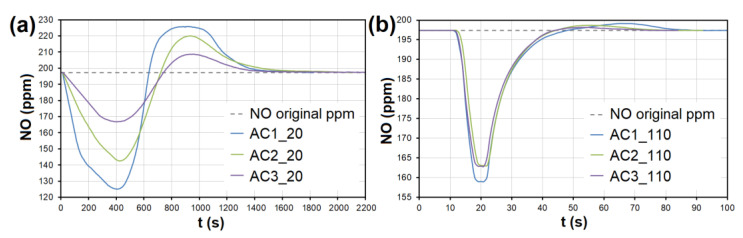
The adsorption curves showing NO adsorption on AC at adsorbent temperatures of (**a**) 20 °C and (**b**) 110 °C. The curves also show forced the desorption effect in the ranges of (**a**) 630–2150 s and (**b**) 43–93 s. AC1-3—activated carbon at different granulometric fractions (see Table 1).

During the adsorption of NO on AC, there was a significant competitive effect between NO and SO_2_ at both temperatures (Figure 7 and Figure 8). As soon as most of the AC adsorption sites were occupied by SO_2_ molecules and the adsorbed amount began to decrease (*t* = 550–950 s at 20 °C, 40–70 s at 110 °C; Figure 7), SO_2_ began to replace NO molecules, resulting in NO forced desorption. The more porous the AC (smaller granulometric fraction), the earlier the desorption effect occurs (represented by AC1, AC2, and AC3 borderlines in Figure 7).

At the temperature of 20 °C, a total of 214.32/92.66 μg/g of NO was adsorbed/desorbed on AC1, 163.92/60.86 μg/g on AC2, and 106.51/30.03 μg/g on AC3 (Figure 8). This phenomenon was investigated in more detail using molecular dynamics (see Section 3.3). Forced desorption was not observed with any other adsorbent.

**Figure 9:**
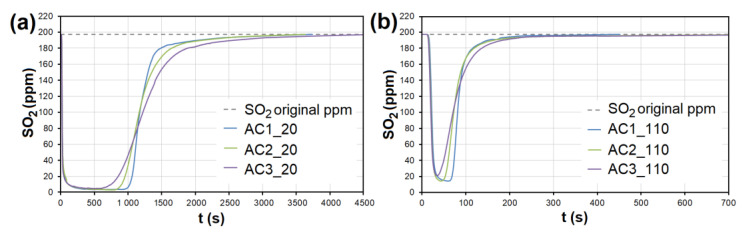
The adsorption curves showing SO_2_ adsorption on AC at adsorbent temperatures of (**a**) 20 °C and (**b**) 110 °C. AC1-3—activated carbon at different granulometric fractions (see Table 1).

**Figure 10:**
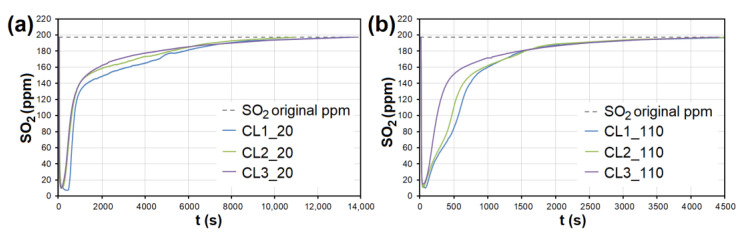
The adsorption curves showing SO_2_ adsorption on CL at adsorbent temperatures of (**a**) 20 °C and (**b**) 110 °C. CL1-3—clay at different granulometric fractions (see Table 1).

**Figure 11:**
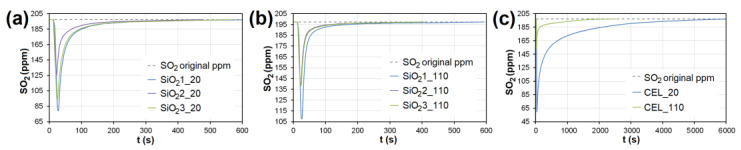
The adsorption curves showing SO_2_ adsorption on SiO_2_ at adsorbent temperatures of (**a**) 20°C and (**b**) 110 °C. (**c**) The adsorbing curve showing SO_2_ adsorption on CEL at adsorbent temperatures of 20°C and 110 °C. SiO_2_1-3—silicon dioxide at different granulometric fractions (see Table 1), CEL—cellulose.

In the case of AC, the adsorption maximum of NO was reached gradually (Figure 8), while the SO_2_ adsorption maximum of SO_2_ occurred immediately after the EG flow started (Figure 9). The same was observed during SO_2_ adsorption on CL, SiO_2_ and CEL (Figure 10 and Figure 11).

### 3.3. Molecular Modeling

**Figure 12:**
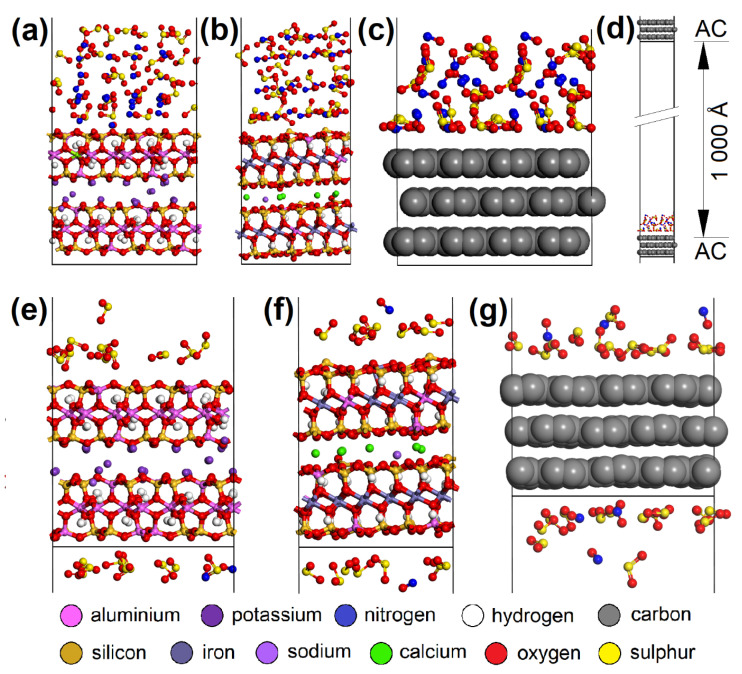
The initial molecular models containing 28 SO_2_ and 28 NO molecules on the (**a**) MUS, (**b**) NON, and (**c**) AC surfaces. (**d**) The initial AC cell with EG molecules and the vacuum slab. The models after molecular dynamics showing the preference of SO_2_ over NO molecules during the adsorption process on the (**e**) MUS, (**f**) NON, and (**g**) AC surfaces. MUS—muscovite, NON—nontronite, AC—activated carbon; EG—emission gas.

**Figure 13:**
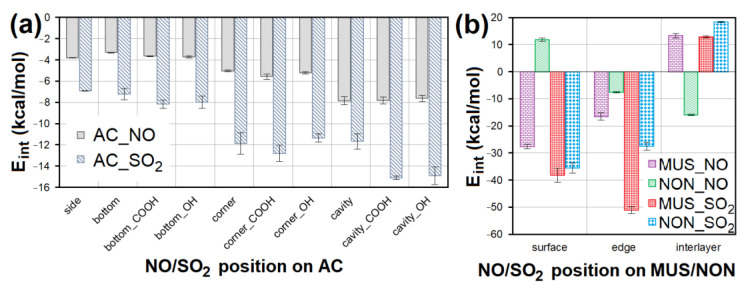
The *E_int_* comparison of models containing NO/SO_2_ at different sites of (**a**) AC surface without or with -COOH/-OH groups and (**b**) MUS/NON surfaces. AC—activated carbon, MUS—muscovite, NON—nontronite, *E_int_*—interaction energy.

CO_2_ adsorption was not observed on any of the adsorbents, and CO was only adsorbed in negligible amounts and only at a temperature of 110 °C. Therefore, these two EG components were not molecularly modeled. Molecular dynamics clarified the competitive adsorption process of NO and SO_2_ on the most adsorbing materials, i.e., AC (having the most specific adsorption process; Figure 12) and CL (MUS and NON). It showed NO forced desorption caused by SO_2_ adsorption. Consequently, it was found with geometry optimization that SO_2_ showed a lower *E_int_* with the surfaces than NO (Figure 13a,b), which correlated with the molecular dynamics results. The SO_2_ molecules gradually occupied the top of the first surface, and the bottom of the second one. According to the notation “EG molecule_EG molecules number on the top of the surface/on the bottom of the surface_in the vacuum slab”, there were SO_2__12/13_3 on AC, SO_2__10/9_9 on MUS, and SO_2__7/7_14 on NON (Figure 12), while most NO molecules were expelled away from the surfaces (NO_3/3_22 for AC, NO_0/2_26 for MUS, and NO_1/1_26 for NON; Figure 12). The molecular dynamics results correspond to the experimentally determined sorption capacities, when higher sorption capacities were measured for SO_2_ than for NO; and at 20 °C (293 K), AC showed higher sorption capacity than CL (MUS/NON; Table 3).

Due to the established correlation of molecular dynamics and geometry optimization results, the other models were only geometry optimized with one EG molecule on the surfaces in order to determine *E_int_* and observe molecular interactions in more detail.

Geometry optimization showed the suitability of the chemical composition of all sorbent materials for NO and SO_2_ adsorption. An attractive interaction with NO or SO_2_ was observed for all models containing AC. Average *E_int_* of models containing NO was 2× higher than models containing SO_2_ on AC (Figure 13a). NO interacted most strongly with the cavities, cavity_COOH (*E_int_* = −8.312 kcal/mol) and cavity_OH. The second strongest NO interactions were observed with the AC corner surfaces (*E_int_* = −5.962 kcal/mol). In the case of SO_2_, the strongest interactions were observed for cavity_COOH (*E_int_* = −15.360 kcal/mol) and cavity_OH. Corner_COOH (*E_int_* = −13.877 kcal/mol) showed the second strongest interactions with SO_2_. These findings prove that the interaction of NO/SO_2_ with AC is stronger, the more numerous surface atoms participating in the non-bonding interaction. The presence of the functional groups (mainly -COOH) is another important factor supporting the strength of AC interaction with NO/SO_2_ via hydrogen bonds.

NO showed the strongest interaction with the MUS surface (*E_int_* = −28.785 kcal/mol) and NON interlayer (*E_int_* = −16.177 kcal/mol). The lowest *E_int_* showed the model containing SO_2_ at the MUS edge (*E_int_* = −52.357 kcal/mol); and the second strongest SO_2_ interaction was with MUS and NON edge (Figure 13b). The MUS and NON interlayers in most cases showed a positive *E_int_* (repulsive force) with NO and SO_2_, due to a large number of cations in the interlayer of clay minerals causing a strong interaction between clay layers, and making it impossible for NO or SO_2_ to penetrate. At the same time, these cations occupied all chemically suitable sites for interaction with NO and SO_2_. The average *E_int_* of models containing SO_2_ on MUS and NON is significantly lower than in the case of models with NO.

**Figure 14:**
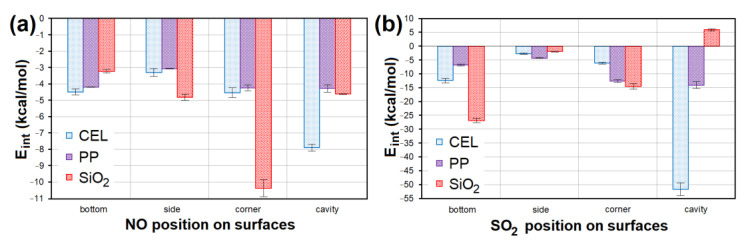
The *E_int_* comparison of models containing (**a**) NO and (**b**) SO_2_ at different sites of CEL, PP and SiO_2_ surfaces. CEL—cellulose, PP—polypropylene, SiO_2_—silicon dioxide, *E_int_*—interaction energy.

All models containing NO placed at all sites of the CEL, PP and SiO_2_ surfaces showed a weak negative interaction energy. The strongest interaction (*E_int_* = −10.936 kcal/mol; Figure 14a) was achieved in the case of NO lying in the corner of SiO_2_. The second lowest *E_int_* (−8.137 kcal/mol; Figure 14a) was achieved for NO placed in the CEL cavity. SO_2_ interacted with CEL, PP and SiO_2_ surfaces several times stronger. The model with the strongest interaction of SO_2_ in the CEL cavity showed 5× lower *E_int_* (−55.491 kcal/mol; Figure 14b) than the model with the strongest interaction with NO. The second strongest interaction (*E_int_* = −27.872 kcal/mol; Figure 14b) was shown by SO_2_ on the SiO_2_ bottom. The average *E_int_* of models containing NO/SO_2_ on PP was the highest.

### 3.4. Adsorption Factors

The importance of chemical (*E_int_*) and textural (*S_BET_*, *S_meso_*, *V_micro_*, *V_net_*) properties on the adsorption capacity of adsorbents was determined (Table 4). AC adsorption capacity was high for both NO and SO_2_, which was mainly correlated with the large surface area in spite of high *E_int_* values (i.e., weak interactions). The high CL adsorption capacity was due to a combination of both chemical and textural properties. A dominant factor of low SiO_2_ and PP adsorption capacity was primarily textural properties (despite strong interactions). The high CEL adsorption capacity was mainly provided by the presence of functional groups strongly interacting with SO_2_ molecules.

Furthermore, agreement between the experiment and molecular modeling was generally more common for lower sorption capacities for NO (corresponding to higher *E_int_* values, i.e., weaker interaction) compared to higher SO_2_ capacities (corresponding to lower *E_int_* values, i.e., stronger interaction).

In general, a significantly lower *E_int_* can be observed for models containing a molecule in a molecular pore (mainly CEL), which appears to be an ideal combination of chemical (more non-bonding interactions) and structural/steric (molecule size correlates with pore size) factors. The agreement of the force field-based molecular modeling results and experimental data demonstrate the modeling strategy used to be a suitable tool for analysis and prediction of interactions during the sorption process at the molecular level.

However, other modeling studies performed using more computationally demanding DFT methods have led to similar results for interactions between AC and NO/SO_2_ [32,34]. Wang et al. [34] reported the same behavior of NO on AC—the strongest interactions were observed on AC basal plane (compare with “side” in Figure 13a), weaker interactions with -OH group (compare with “bottom_OH” in Figure 13a), and the weakest with -H (compare with “bottom” in Figure 13a). Zhao et al. [32] found the same *E_int_* trend for SO_2_ on AC, where the molecule interacted most strongly with -COOH groups (compare with “bottom_COOH” in Figure 13a), more weakly with -OH (compare with “bottom_OH” in Figure 13a), and the weakest with -H (compare with “bottom” in Figure 13a).

The comparison demonstrates the usefulness of computationally less demanding force field-based molecular modeling in adsorption research. It also reveals that there is no need to take into account the partial amorphousness of AC, e.g., by a large amount of polyaromatic hydrocarbons [32]. Approximation of AC using graphite, which additionally enables the preparation of pores of the required size, is sufficient for the given purpose.

## 4. Conclusions

In this work, the competitive adsorption of emission gas, containing CO, NO, SO_2_ and CO_2_, on adsorption materials with different chemical compositions was studied.

CO_2_ was not adsorbed on any material. CO was adsorbed in small amounts and mainly at a temperature of 110 °C on AC, CL and CEL. At 20 °C, the AC showed the highest NO and SO_2_ sorption capacity. At 110 °C, the highest NO and SO_2_ sorption capacity was found for the CL.

The strongest interactions were exhibited between NO/SO_2_ and corner or cavity surface types due to the larger number of interacting atoms. Carboxyl and hydroxyl functional groups strengthened the interaction of AC mainly with SO_2_, where hydrogen bonds were formed. NO and SO_2_ showed attractive interactions with MUS and NON mainly on the surface and edge. Strong interactions between clay layers and interlayer cations prevented the penetration of NO and SO_2_ into the interlayer space.

The dominant factor determining the sorption capacity of AC, SiO_2_ and PP was textural (surface area, porosity)—while in the case of CEL, it was the chemical factors (chemical composition, functional groups). Both textural and chemical factors played an important role in adsorption on CL.

The effects of competitive adsorption of emission components on sorption capacity were determined and further clarified using force field-based molecular modeling. The modeling strategy made it possible to simulate surfaces with different and controllable structural character. Geometry optimization and molecular dynamics results agreed well with experimental data allowing the modeling strategy to be further used in studies of interactions during adsorption, or for a prediction of adsorption phenomena.

## Figures and Tables

**Table 1:** Basic information about the adsorbing materials used, such as particle density and material humidity, and its forms and labeling.

Adsorbing Material	Particle Density (g/cm^3^)	Humidity (%)	Adsorbing Material Form	Granulometric Fraction Labels		
				0.16–0.315 mm	0.315–0.63 mm	0.63–1.00 mm
AC	1.354	3.318	powder	AC1	AC2	AC3
CL	2.214	11.992	powder	CL1	CL2	CL3
SiO_2_	1.754	0.285	powder	SiO_2_1	SiO_2_2	SiO_2_3
CEL	1.225	15.339	pellets	-	-	-
PP	1.007	1.204	nonwoven	-	-	-

AC—activated carbon, CL—clay, SiO_2_—silicon dioxide, CEL—cellulose, PP—polypropylene.

**Table 2:** Determined textural properties of adsorbents.

Adsorbent	*S_BET_* (m^2^/g)	*S_meso_* (m^2^/g)	*V_micro_* (mm^3^liq/g)	*V_net_* (mm^3^liq/g)
AC1	999	209	373	542
AC2	975	180	376	521
AC3	868	114	359	455
CL1	76	49	14	134
CL2	78	50	14	131
CL3	70	47	12	127
SiO_2_1	8.9	8.9 *	n. d.	41
SiO_2_2	6.4	6.4 *	n. d.	35
SiO_2_3	8.5	8.5 *	n. d.	45
CEL	1.6 ± 0.1	n. d.	n. d.	n. d.
PP	0.4 ± 0.1	n. d.	n. d.	1.02

* Since adsorbent belongs to meso–macroporous solids, the mesopores surface area *S_meso_* equals the specific surface area *S_BET_*. AC1-3, CL1-3, SiO_2_1-3—activated carbon, clay, and silicon dioxide, respectively (1-3—three granulometric fractions; see Table 1), CEL—cellulose, PP—polypropylene; *S_BET_*—specific surface area, *S_meso_*—mesopore–macropore surface area, *V_micro_*—micropore volume, *V_net_*—net pore volume, n. d.—not defined.

**Table 3:** The adsorbents in the wet and dried state and their adsorption capacities for different molecules (NO, SO_2_, CO) at different adsorbent temperatures (20 and 110 °C).

Adsorbent	Mass		Adsorption Capacity				
	Wet Sample (g)	Dried Sample (g)	NO		SO_2_		CO
			20 °C (μg/g)	110 °C (μg/g)	20 °C (μg/g)	110 °C (μg/g)	110 °C (μg/g)
AC1	1.5277	1.4770	120.83	2.27	3549.61	190.00	0.47
AC2	1.5370	1.4860	102.23	1.85	3482.41	182.27	0.17
AC3	1.5404	1.4893	75.65	2.01	3724.97	186.58	0.22
CL1	2.8859	2.5398	20.28	6.20	2771.37	1182.46	5.69
CL2	2.7871	2.4529	8.16	4.53	2281.26	1110.76	4.86
CL3	2.7690	2.4369	4.98	3.43	2242.24	888.29	3.80
SiO_2_1	1.6116	1.6070	2.11	-	71.68	28.01	-
SiO_2_2	1.6741	1.6693	0.33	-	68.42	5.95	-
SiO_2_3	1.5494	1.5450	0.10	-	59.72	4.69	-
CEL	1.6520	1.3986	5.09	1.13	1141.61	154.89	1.48
PP	0.7907	0.7812	-	-	-	-	-

AC1-3, CL1-3, SiO_2_1-3—activated carbon, clay, and silicon dioxide, respectively (1-3—three granulometric fractions; see Table 1), CEL—cellulose, PP—polypropylene.

**Table 4:** Determining the importance of *E_int_* or textural properties (*S_BET_*, *S_meso_*, *V_micro_*, *V_net_*) on adsorption capacity of the adsorbents having 0.16–0.315 mm granulometry.

Adsorbing Material	Adsorption Capacity (μg/g)		Lowest *E_int_* (kcal/mol)		*S_BET_* (m^2^/g)	*S_meso_* (m^2^/g)	*V_micro_* (mm^3^liq/g)	*V_net_* (mm^3^liq/g)
	NO	SO_2_	NO	SO_2_				
AC	120.83	3549.61	−8.31	−15.36	999 *	209 *	373 *	542 *
CL	20.28	2771.37	−28.79 *	−52.36 *	76 *	49 *	14 *	134 *
SiO_2_	2.11	71.68	−10.94	−27.87	8.9 *	8.9 *	-	41 *
CEL	5.09	1141.61	−8.14 *	−55.49 *	1.6 ± 0.1	-	-	-
PP	-	-	−4.60	−16.23	0.4 ± 0.1 *	-	-	1.02 *

* denotes dominant adsorption factor, i.e., chemical (*E_int_*) or textural (*S_BET_*, *S_meso_*, *V_micro_*, *V_net_*). AC—activated carbon, CL—clay, SiO_2_—silicon dioxide, CEL—cellulose, PP—polypropylene, *S_BET_*—specific surface area, *S_meso_*—mesopore–macropore surface area, *V_micro_*—micropore volume, *V_net_*—net pore volume, n. d.—not defined, *E_int_*—interaction energy.

## Data Availability

Data is contained within the article.
